# Ten simple rules for biologists initiating a collaboration with computer scientists

**DOI:** 10.1371/journal.pcbi.1008281

**Published:** 2020-10-22

**Authors:** Monika Cechova

**Affiliations:** Central European Institute of Technology (CEITEC), Genetics and Reproductive Biotechnologies, Veterinary Research Institute, Brno, Czech Republic; Dassault Systemes BIOVIA, UNITED STATES

Biology is increasingly digital, and scientists are generating huge amounts of data daily, turning molecules into sequences and text files. As a biologist, you might need help analyzing all these data and have considered collaborating with a computer scientist for the first, second, or third time. This person might have some training in computational biology, but their main focus has always been computer science (CS), and here is the challenge: how do you talk to them? They might be able to write efficient code, but they often do not know some of the basics of biology. When they look at your molecules, some of them might see text files before biology. Also, if explaining things takes so much time, is it worth it? Should you be analyzing your own data instead? Or perhaps you have noticed that all those big, shiny papers of today represent a smart blend of biology and CS. You have found a collaborator and want to learn how to engage them. These 10 simple rules aim to help.

## Rule 1: Do not try to turn them into biologists

You are likely overestimating the level of understanding needed for the collaboration to be successful. Your job is not to teach computer scientists what you learned in your many years of studying biology. Your job is to provide them intuition to think about the problem the right way and to motivate them to ask questions. There are multiple ways to achieve this. First, draw concepts. My first mentor in science, Eduard Kejnovsky, has somehow always had a pen and a paper around and explained everything in drawings. This has been incredibly helpful because those drawings turned into images I could remember. Second, always provide a range of reasonable values to ground the analysis. If it is not okay to have thousands of chromosomes, then what are those sequences? What allele frequencies should you expect to observe in the variant call format (VCF) file you downloaded? What is a gene, and what length distribution and intron distribution do you expect to see? In other words, you as a biologist have a very good intuition for “how much of something” makes sense or not. Pass this intuition along, because this is not something a computer scientist can easily look up or read upon. Define the extreme values that can be expected in the data. Help computer scientists turn this intuition into unit tests (when small parts of code are being tested) with warnings. At the very least, your pipelines need to report any unusual observations they catch in the data, whereas the definition of unusual must be provided by a biologist. Revisit your definitions as the project progresses to make sure there is a mutual understanding.

## Rule 2: Do not judge knowledge gaps

Even the lack of most basic knowledge is okay. People can live happy lives without ever knowing what a centromere is or understanding the difference between meiosis and mitosis. If you encounter an important gap in knowledge of your collaborator, pause for a few seconds, and think about the best way to communicate this concept. Do not act surprised, do not act disappointed, and do not look bothered. Simply and calmly explain the concept. Finish your explanation with questions like “does this make sense?” and “what questions do you have?”. They will return the favor when it will be time to talk about CS. Remember that you are collaborating to complement each other, not to compete, so the fact that your collaborator does not have a deep knowledge of your subject might actually be a good thing; it enabled them to become an expert in theirs.

## Rule 3: Learn how computers store data and format information in a computationally friendly way

Just because you have downloaded a file does not mean it is downloaded correctly. The integrity of the file could be compromised for a multitude of reasons: the disk might have run out of space or the network connection might have broken for an instant. One way to make sure the file is intact is by using so-called hashing. Instead of looking at the whole file, we generate a description of a file (typically in the string of letters and numbers). If 2 files are the same, they will be described in the exact same way. If 2 files are different, it’s very, very unlikely they could be accidentally described the same way. The popular way of hashing is running md5sum (md5 on Mac), just type “md5sum filename”. What about that huge fastq file you have? Try changing a “single” character and compare if md5sum is still the same (it is not). Use this whenever you download data from a database or whenever you send or receive data from a collaborator. For a computer scientist, 2 files are the same if they are identical. Play along. When copying files, learn to use rsync instead of cp/mv so you don’t have to worry about closing your laptop or a power outage as this command can successfully continue after an interruption.

Initially, it might not be intuitive to format your data in a way that works well with computational pipelines and software packages, but there are some conventions you can follow. For example, your group should agree on numbering chromosomes either as numbers or the combinations of letters and numbers (1 versus chr1). This is a source of innumerable misunderstandings in bioinformatics, especially when rerunning pipelines that worked perfectly fine a week ago but somehow do not work with the new data. Be careful with lowercase, uppercase, and singular versus plural naming schemes. Again, whether your sequenced tissue originates from testis or testes might make a little difference to you but will confuse your collaborator.

Missing data are not necessarily missing. There is a difference between an empty cell that can be empty for a number of reasons and a truly missing data point—when we just do not know. Perhaps our collaborators did not note whether our sample is a male or a female individual or exactly from which tissue they extracted the DNA. This is very different from a cell that is empty because we have not filled that information yet, conceivably because we will find out that information in the future. Also, do we even need to know this information at all? These are many different scenarios marking information in a different way that might become important during an analysis. For example, many scientists combine sequencing data sets, but do all these data sets originate from the same individual? This might become important when you see subtle differences in your joint data because some of the variation might be due to technical and some due to biological reasons. Therefore, develop strict guidelines, and be consistent. Marking missing data with NA,–, or, worst of all, blank space might all mean the same thing to you, but they mean the same thing neither for your computer nor for the computer scientists you are collaborating with. Yes, collecting data on humans, in particular, might get messy, and the collected information will never be perfect, but there is no reason why your notes should not be!

Lastly, what is friendlier than providing examples? All requests on the side of a biologist must come with examples or test data, meaning the expected output for a given input. This does not need to be formal; although that is ideal, you could just provide a list of genes that you expect to see in the output and list of genes you never expect to see in the output, as a control. Computer scientists will turn these examples into tests that can be run repeatedly. This will ensure that even after many modifications of the code, specific input files will still yield specific output files.

To conclude, picking a consistent naming scheme, meticulously documenting missing data, and providing test data sets are all good steps toward “computational friendliness.”

## Rule 4: Describe your data in a way that facilitates collaboration

There is a whole field of CS dealing with how to store information in databases, yet there are things you can do now to facilitate reusing your data by others. First, if you store your data in rows, then all your tables need to have a column with a unique identifier or a key, and this column needs to be universally unique. If your sample is named orangutan, what will happen once you sequence data from another orangutan? Dates can also be helpful, for example, marking a sequencing run from 10/06/2020 as such, but even here you need to be careful as time stamps are understood differently in different regions. The example above would likely be understood as sequenced on June 10, 2020 in the EU region, but in North America, it could be read as October 6, 2020. If there is no ID accompanying your sample, create it yourself. This way, you can recognize all the places where this particular sample has been used.

Your tables should be described in a way that requires the “least amount” of expert knowledge to understand them. If all of your samples have a column describing whether they are DNA or RNA, do not write anything else in that column! You might be tempted to write “cell culture” or “flow-sorted material,” since for you it is obvious these are DNA samples, but for your collaborators, this will not be obvious. Even worse, when they will use this column to shortlist all samples with DNA, they will miss these special rows. Correspondingly, if I was writing “10 simple rules for computer scientists to collaborate with biologists,” I would say that biologists write unpredictable things into cells of their tables! It is essential to look at the summary of all the values before pulling anything out of the table. It is also important not to duplicate information and to use consistent separators, as mixing tabulators, commas, and other symbols is rarely a good idea.

In summary, describe your data in a logical, coherent way and make sure no insider or expert knowledge is needed for your collaborator to start working with these data.

## Rule 5: Learn if you should speed up your code (and how)

“Premature optimization is the root of all evil” [1] goes one of the most famous quotes in CS. While there are things where you shouldn’t compromise, such as documenting your code, is it really necessary for your script to finish in 5 minutes instead of 15? How often will this script be run and by how many people? How much time will they all cumulatively save if you speed it up? Before optimizing anything, I recommend checking this brilliant guide by xkcd [2]. Note that this guide assumes it is your time you are optimizing, not an algorithm, but it’s a good starting point to provide you an intuition about the trade-off.

Let’s say that you decided that you do want to speed up your code. Some parallelization is natural and rather straightforward, such as running your script by chromosome. In such instances, there is typically no reason “not” to run it in parallel if your underlying data are statistically independent. Another option is to make your code work with multiple central processing units (CPUs), but I would not recommend this as a first measure because, when done poorly, it might compromise the integrity of the results. For many applications, splitting data into batches and running them all in parallel with the same script and afterward combining the results is a good option.

As a biologist, you probably also heard that some programming languages are faster than others. However, in day-to-day science, I strongly believe that readability and reproducibility come before speed. Other scientists might be more comfortable reusing your high-level Python code compared to a faster low-level C code. Note that here, I am not talking about software that will be run thousands of times, potentially by as many users. I am referring to code that accompanies your research findings, which will more likely than not be a combination of established pipelines and custom scripts. Therefore, do not engage in a collaboration with the aim of speed up without a careful thought.

## Rule 6: Think about concepts and the audience, not the programming language

At its heart, CS is about concepts and abstractions, not about any of the programming languages. Many computer scientists do not really care about the implementation in any particular language. When collaborating with computer scientists, do not push for your ideas of implementation. Instead, communicate the scope of the product you want to see and the concepts behind it. Biologists sometimes get too attached to certain solutions (C++ is fast, SQL is necessary) that it might be challenging for them to step back and instead think about their original goals.

You probably heard that “off-by-one error” is one of the most common errors in bioinformatics. Why? One of the reasons is that biologists typically start counting with 1, while computer scientists are trained to start with 0 (so that second position is pointing to the third element). In order to effectively collaborate, you need to learn the differences between 0-based and 1-based intervals, half-open and open intervals, and anytime you are sharing any types of interval data, communicate which one you are using. Commonly used formats for intervals are .bed (0-based) and .gff (1-based), and you could even choose to only use 1 of these for a specific project. Similarly, 2 popular programming languages, Python and R, also tend to start numbering with 0 and 1, respectively.

In conclusion, pick a problem over technology, but when you do pick a technology, learn its caveats.

## Rule 7: Aim for transparency and reproducibility, share your code at every stage

Do not wait for the final product before you start collaborating with computer scientists or dare to show them your pipelines. Many biologists are conscious about sharing their code and typically try to delay sharing until the very last, prepublication moments. Yet, this is too late. The code can be stored in special storage—named repository—and be made accessible “immediately” within a private group. Private repositories are typically free for academic institutions and can be incrementally improved up until the publication date when switched to public. Some scientists even choose to open their code immediately to stimulate collaboration and to have tangible evidence of their idea in the public domain. Sometimes, the reason not to share is embarrassment over how “imperfect” the code is. Virtually, all biologists feel this way when sharing their code with computer scientists. Code review (somebody else going over your code and pointing out inconsistencies and errors) should not be an uncomfortable practice, but rather a standard process like in CS and engineering. Learn to be grateful that someone is willing to look at your code and test it. You wouldn’t publish a paper in which you were the only person ever to read it, why would it be okay with a script?

Do not send your code by email; instead, provide a link to the repository you are using and refer to the specific versions by commit IDs or dates, so that you do not have a folder with 50 different versions of the code you are writing. Nowadays, submitting code to a repository can be conveniently done with a graphical interface in your browser, so you do not even need to open a terminal to do that. When in doubt, ask your collaborators for help. A multitude of tools can be used for the collaboration to go more smoothly, such as git (GitHub (https://github.com) or Bitbucket (https://bitbucket.org)), Jupyter (https://jupyter.org; iPython, https://ipython.org) notebooks, Rmarkdown (https://rmarkdown.rstudio.com), Galaxy (https://galaxyproject.org), snakemake (https://snakemake.readthedocs.io), nextflow (https://www.nextflow.io), and LaTeX (https://www.latex-project.org) (and Overleaf; https://www.overleaf.com). If these sound overwhelming and you are unsure whether investing time in learning any of them is worth it, ease into it by attending (online) workshops or seminars or paying attention when somebody else mentions them.

While all of these tools will make your research more reproducible, note that the true reproducibility is not about getting a very specific output for a very specific input using a defined pipeline. Paraphrasing István Albert, true biology should be rather agnostic to software versions or minor decisions about parameters or the random seed [3], and ideally, the results should be later validated on an independent data set to avoid a multiple testing problem [4].

In summary, share your code early, openly, and often.

## Rule 8: Understand strengths and limitations of each discipline

CS and biology are different in how they handle abstraction and complexity [5], in how they approach problems, and also “which” problems they are interested in. CS faculty and CS students are not usually doing CS research to help people solve their biological research problems. Rather, they are hoping to invent new CS, just as biologists are hoping to discover new biology. The biologist should understand what the computer scientist is aiming to get out of this collaboration—and it is typically not the chance to do a bit more programming or to answer their biological question. Just like running yet another polymerase chain reaction (PCR) is not particularly rewarding for a biologist, writing yet another short script converting between data formats is not particularly rewarding for a CS person. A novel piece of CS implemented in the form of a tool can be published simultaneously with a biological paper applying it to real data. Note that computer scientists publish differently, mostly in the form of conference proceedings, which are (typically rather concise) communications advancing the field. To the contrary, biological papers tend to have many coauthors providing diverse expertise and sometimes excessive supplemental material.

Computer scientists have a long tradition of open sourcing, while biologists often rely on proprietary methods and knowledge. These worldviews clash in discussions on “how” and “when” data, protocols, and code should be shared. Computer scientists often have an inclination toward the concepts of open science; as Martin Čech once said, “You cannot build a community behind closed doors.”

Biologists routinely deal with experimental errors (and “noisy” data), while computer scientists typically have limited experience dealing with these. Thus, it is advantageous to communicate the definitions of biological and technical replicates for any given experiment, as well as the interpretation of false positives and false negatives. Computer scientists are better trained to formally define the conditions and limitations of each experiment and model and should be given a space to do so, whenever possible.

Establishing both shared and individual goals of a biologist and a computer scientist, understanding strengths and limitations of each discipline, accompanied by an understanding and acceptance, as well as developing a plan of how each contribution will be credited, goes a long way toward a collaboration and is essential to sustain it in the long run.

## Rule 9: Communicate how the results of your collaboration will be validated

What will be the outcome of your collaboration? How will you validate the data that the computer scientist will produce? This is important to establish early so that the collaborators have a clear idea about the possibilities and feasibility of wet lab validation. Are you interested in just a handful of high-confidence candidates? Are you able to validate hundreds of findings? Discussing this might also increase the personal investment in the collaboration, as validating one’s findings is typically satisfying for computer scientists.

Just like writing is an iterative process of improvement, so is the joint project development with a computer scientist. In each iteration, the pipeline is refined until the satisfactory output is achieved and, hopefully, reproducible. Initially, biologists might not even be able to fully define the parameters of the pipeline they are requesting but will guide their decisions based on the data. Other times, the intermediate results will need to be validated, which will take time and effort. Rarely will an initial pipeline suffice for all the future applications, so the long-term collaboration is favorable.

Quality control is an essential part of every pipeline and needs to be developed jointly among the biologist and computer scientist, to account for both expected biological variation and possible errors during data processing (e.g., mishandling of forward/reverse strand). Some additional experiments might be suggested by a computer scientist in order to calibrate the pipeline and will seemingly offer little benefit for the biologist. Other times, the biologist might choose to blind the computer scientist to the tested hypothesis in order to not bias them toward a particular result.

Finally, because pipelines can be improved indefinitely, emphasizing the back-and-forth nature of the progress, communicating the characteristics of the final product, and discussing the types of validation that will be performed are all good ways to set initial expectations.

## Rule 10: Do not get involved in wars over what the “real” science is

This is probably the most important rule of them all. Things that you can touch and manipulate, e.g., poke with a stick or look under a microscope, are amazing. Yet, there are whole labs that only do in silico biology. When the late James Taylor joined the Biology department at Johns Hopkins University (JHU), the first thing he did was remove the unnecessary wet lab benches from his new lab space. He didn’t need them to become a champion of reproducibility and open science. Science is no more real if it happens on the computer or the bench. We are all so used to working with things we cannot see or touch, why would computationally driven biology be an exception?

This issue is important because computer scientists that are not perceived as doing real biology cannot be credited as such. A biologist generates the data, yet there is a need for computational knowledge in order to analyze the data. Without biological expertise, there would be no data; without computational expertise, there would be no analysis; but precisely, this is the beauty of collaboration that is luckily being increasingly recognized by shared first authorships. While you can certainly outsource some typical computational problems (think differential gene expression analysis or a genome assembly), it is essential to treat your collaborators differently. Computer scientists don’t work for you, but with you. Recognize the value of interdisciplinary research instead.

Interdisciplinary collaboration can be frustrating, tedious, and full of misunderstandings. However, it can also be the most amazing and most satisfying scientific journey—“if” you combine a group of people with the right attitude toward learning and from complementary backgrounds. Computer scientists will ask you mind-blowing questions that you have never thought of. This is because they did not learn biology in a stepwise multiyear learning process as you did. Their knowledge is shattered into thousands of loosely connected pieces, and it is specifically when they zoom in to those loose connections when they will ask you questions to which no one in the field knows answers. It’s a wild and rewarding ride and the one you can prepare for. Hopefully, these 10 simple rules will help you buckle up to take it.
